# Menopause symptom prevalence in three post–COVID-19 syndrome clinics in England: A cross-sectional analysis

**DOI:** 10.1016/j.ijregi.2024.100405

**Published:** 2024-07-15

**Authors:** Stuart Stewart, Adrian Heald, Yvette Pyne, Nawar Diar Bakerly

**Affiliations:** 1 Donal O'Donoghue Renal Research Centre, Northern Care Alliance NHS Foundation Trust, Salford, United Kingdom; 2 Centre for Primary Care & Health Services Research, University of Manchester, Manchester, United Kingdom; 3 Rochdale Care Organisation, Northern Care Alliance NHS Foundation Trust, Rochdale, United Kingdom; 4 School of Medical Sciences, University of Manchester, Manchester, United Kingdom; 5 Salford Royal Hospital, Northern Care Alliance NHS Foundation Trust, Salford, United Kingdom; 6 Centre for Academic Primary Care, University of Bristol, Bristol, United Kingdom; 7 School of Biological Sciences, Manchester Metropolitan University, Manchester, United Kingdom

**Keywords:** Menopause, Perimenopause, Women's Health, COVID-19, Long COVID, Population health

## Abstract

**Objectives:**

There is an overlap between symptoms of perimenopause/menopause and post-COVID syndrome and there is a concern that some female patients referred to post-COVID syndrome clinics may have undiagnosed perimenopause/menopause. However, the prevalence of such symptoms in this population is unknown.

**Methods:**

Cross-sectional analysis of 122 women's health questionnaires as part of a service improvement project in three National Health Service post-COVID syndrome clinics in England. The primary outcomes were prevalence of menopause symptoms and association between the total menopause symptom questionnaire (MSQ) score and the key predictors.

**Results:**

Age group 40-54 years showed the highest prevalence of most individual symptoms and the highest mean total MSQ score (36.4; confidence interval [CI] 32.3-40.6), correlating clinically with the onset of perimenopause/menopause. Regression modeling shows a significant positive parabolic relationship between age and total MSQ score. Age group 40-54 years is associated with a 6.60-point higher (CI 1.31-11.9) total MSQ score than the age group 55-79 years; an increase of one index of multiple deprivation quintile is associated with a 2.85-point lower (CI −1.24 to −4.45) total MSQ score; presence of a gynecologic diagnosis is associated with a 6.31-point higher (CI 1.32-11.3) total MSQ score. A total of 51% of patients who menstruate reported menstrual disturbance with COVID-19 infection and 21% with COVID-19 vaccination.

**Conclusions:**

Symptoms possibly attributable to perimenopause and menopause were highly prevalent among female patients attending post-COVID syndrome clinics in Greater Manchester, England. Our findings provide key prevalence estimates and significant predictors of MSQ scores that are vital for future research, clinical practice, and policy.

## Highlights

• Possible menopause symptoms are prevalent in women in three post-COVID syndrome clinics.
• Possible menopause symptoms are generally higher than existing data.
• Higher symptom prevalence may reflect overlapping burden of post-COVID syndrome.
• Age and socioeconomic deprivation predict total menopause symptom score.
• Presence of any gynecologic diagnosis may also predict menopause symptom score.

## Introduction

Post-COVID syndrome is a multisystem disorder after infection with SARS-COV-2, with over 200 symptoms documented across numerous studies [1]. Such a vast array of clinical manifestations stimulated the development of a clinical case definition by a Delphi consensus in 2022 [2]. Although our understanding of the pathophysiologic mechanisms to underpin post-COVID symptomatology is evolving, sex-specific research is scant [1], ultimately limiting our understanding of why female sex is associated with a potentially increased risk of post-COVID syndrome [1,3].

Temporary menstrual disturbance observed after acute COVID-19 infection stimulated hypotheses of potential sex organ dysfunction caused by direct infiltration of the SARS-CoV-2 virus due to the high expression of angiotensin-converting enzyme 2 receptors on ovaries and the endometrium [4,5] and multisystem consequences of acute infection [4]. Furthermore, temporary menstrual disturbance measured after COVID-19 vaccination raised the possibility of an immunologic etiologic mechanism [6]. However, distinct mechanisms in COVID-19 infection– and vaccination–associated menstrual disturbance are yet to be determined. Although a temporary disruption of sex hormone synthesis could theoretically exacerbate the symptoms of perimenopause and menopause, there is also a clear overlap between the symptoms of perimenopause/menopause with symptoms of post-COVID syndrome (Table 1). In fact, 14 symptoms (out of 23) on a menopause symptom questionnaire (MSQ) [7] used in National Health Service (NHS) care pathways [8] are also listed as symptoms of post-COVID syndrome in recent and robust observational research of 242,712 patients in England [3] (Table 1).

**Table 1:** Mapping of menopause symptom questionnaire to symptoms of post-COVID syndrome.

Mapping to menopause symptom questionnaire [7]	Post-COVID syndrome symptoms from Atchison et al. [3]	Resolved prevalence reported in Atchison et al. [3] (%)	Ongoing prevalence reported in Atchison et al. [3] (%)
1. Heart beating quickly or strongly	Heart issues (racing heart, palpitations, irregular heartbeat)	14.1	25.2
2. Feeling tense or nervous	Does not map to resolved or ongoing symptom of post-COVID syndrome in Atchison et al*.* [3]		
3. Difficulty sleeping	Difficulty sleeping	39.5	49.8
4. Memory problems	Poor memory	24.1	43.1
5. Attack of anxiety or panic	Anxiety	28.8	39.8
6. Difficulty in concentrating	Difficulty thinking or concentrating	31.9	54.9
7. Feeling tired or lacking in energy	Severe fatigue	4.4	13.1
	Mild fatigue	49.7	66.9
8. Loss of interest in most things	Low mood	25.6	37.9
9. Feeling unhappy or depressed			
10. Crying spells	Mood swing	15.8	25.9
11. Irritability	Does not map to resolved or ongoing symptom of post-COVID syndrome in Atchison et al. [3]		
12. Feeling dizzy or faint	Does not map to resolved or ongoing symptom of post-COVID syndrome in Atchison et al. [3]		
13. Pressure or tightness in head	Does not map to resolved or ongoing symptom of post-COVID syndrome in Atchison et al. [3]		
14. Tinnitus	Hearing issues (hearing loss, tinnitus)	19.5	26.9
15. Headaches	Headaches	34.4	49.0
16. Muscle and joint pains	Aching or cramping muscles, pain in muscles	33.2	48.2
17. Pins and needles in any part of the body	Numbness or tingling somewhere in the body	14.9	25.6
18. Breathing difficulties	Shortness of breath, breathlessness, wheezing	14.4	33.3
19. Hot flushes	Does not map to resolved or ongoing symptom of post-COVID syndrome in Atchison et al. [3]		
20. Sweating at night	Does not map to resolved or ongoing symptom of post-COVID syndrome in Atchison et al. [3]		
21. Loss of interest in sex	Does not map to resolved or ongoing symptom of post-COVID syndrome in Atchison et al. [3]		
22. Urinary symptoms	Does not map to resolved or ongoing symptom of post-COVID syndrome in Atchison et al. [3]		
23. Vaginal dryness	Does not map to resolved or ongoing symptom of post-COVID syndrome in Atchison et al. [3]		

Such overlap has the potential to create diagnostic uncertainty and lead to underdiagnosis of perimenopause/menopause and possibly misdiagnosis of post-COVID syndrome [9]. This is especially important considering a clinical case definition of post-COVID syndrome states it “cannot be explained by an alternative diagnosis” [2].

Despite the symptoms of perimenopause and menopause being easily measured using a MSQ there are no data on the prevalence of these symptoms in women attending post-COVID syndrome clinics nationally or globally; such data are urgently required for several reasons. First, measuring the prevalence of symptoms of perimenopause/menopause in post-COVID syndrome clinics is an urgent equity imperative that is vital to fill in key information gaps in a burgeoning women's health crisis and supported by international calls for research in menopause care [5,10]. Second, measuring the prevalence of symptoms of perimenopause/menopause in post-COVID syndrome clinics will provide essential epidemiologic data for understanding the potential size of the problem, which would inform local and national clinical pathway development, service provision, and, ultimately, policy. Third, it will provide baseline data to help support clinicians in confidently interpreting MSQ results in patients in post-COVID syndrome clinics. Finally, it will provide evidence of the degree of overlapping symptoms, which may help shape our understanding of possible pathophysiologic mechanisms vital for future observational and interventional research.

The primary aim of this study is to measure the prevalence of symptoms of perimenopause/menopause in female patients attending three post-COVID syndrome clinics in Greater Manchester, England and the impact of the key independent variables on symptom scores. The secondary aim is to measure the prevalence of menstrual disturbance associated with COVID-19 infection and vaccination.

## Methods

### Study design

This is a cross-sectional analysis of patient data as part of a service improvement project undertaken within three post-COVID syndrome clinics within the Northern Care Alliance NHS foundation trust (Salford Royal NHS hospital clinic, Heywood-Middleton-Rochdale clinic, and Fairfield clinic), England, UK from February 2023 to May 2023. This service improvement project was agreed with service leads and clinic staff and authorised via the post-COVID syndrome multidisciplinary team within the Northern Care Alliance in October 2022 (ref number: 2023/12). In line with the Northern Care Alliance and Health Research Authority guidelines [11] for service improvement projects, ethics approval was not required because these anonymized survey data were collected as part of routine clinical care used for service improvement.

### Participants

All new female patients aged 18-79 years, identified by their registered sex in the electronic health record, attending one of the post-COVID syndrome clinics, were asked to complete a women's health questionnaire (appendix 1).

### Procedures and outcomes

Recognizing the vast array of symptoms associated with post-COVID syndrome, for the purpose of this study, we define post-COVID syndrome using the symptomatology outlined in the work of Atchison et al. [3]. All eligible patients were asked to complete a women's health questionnaire, which was designed in two parts: part 1 included a range of questions designed to elicit a patient's past medical and surgical histories relating to their gynecologic health, and part 2 included (with permission from Newson Health Ltd.) a MSQ (Balance MSQ [7]), approved by Organisation for the Review of Care and Health Apps (ORCHA) [12], and used within the NHS care pathways [8] to measure menopause symptom experience and prevalence. The Balance MSQ [7] is a variant of the internationally validated Greene Climacteric Scale [13] and was chosen due to its approval by ORCHA and integration in real-world clinical pathways in the NHS, as well as its important measurement of symptoms related to memory changes, hearing, and genitourinary symptoms of menopause. Staff in the post-COVID syndrome clinics undertook all data collection. Patients who did not complete the MSQ component of the overall questionnaire were excluded. Over the study period, 168 new patients were seen across the three clinics, with 122 patients completing the questionnaire in full, representing a response rate of 72.6%.

The following variables were captured in the questionnaire: date of birth, NHS number, clinic location, past experience of menopause symptoms, family history of early menopause, start of menopause symptom experience, menstrual status, menstrual regularity, menstrual cycle length, contraception use, history of gynecologic diagnoses and surgery, menstrual disturbance with COVID-19 infection and COVID-19 vaccination, and the MSQ [7] results. Postcode was captured from the electronic health record to calculate indexes of multiple deprivation (IMD) quintiles, a measure of geographical area level deprivation at a low geographical level of approximately 1600 people, measured in 2019 over several domains (income; employment; education, skills, and training; health deprivation and disability; crime; and housing) [14]. Age was modeled as a continuous numerical variable and transformed into a categorical variable of age groups (18-39, 40-54,and 55-79 years), with analyses conducted across age groups instead of reproductive status because menstrual disturbance with COVID-19 infection and vaccination is a significant confounder, which could result in more patients being considered as perimenopausal. Age group ranges (18-39, 40-54, and 55-79 years) were selected based on preexisting age groups routinely used in clinical practice to demarcate potential menopause diagnoses; menopause earlier than age 40 years is the least common and clinically defined as premature ovarian insufficiency (POI) [15]; menopause between 40-45 years is defined as early menopause [15]; menopause after age 45 years is considered normal, with most women experiencing menopause at age 45-55 years [16]; finally, all women over 55 years are, therefore, likely to have experienced menopause, which may also be defined as post-menopause [16]. All study variables are documented in the data dictionary (Appendix 2).

The primary outcomes were menopause symptom prevalence as measured using the MSQ [7] and quantification of predictor estimates on total MSQ score. On the MSQ, the presence of symptoms was defined as a score of ≥1 and the absence of symptoms was defined as a score of zero. Prevalence was calculated based on the presence of symptoms with score of ≥1 on the MSQ. Secondary outcomes include menstrual disturbance associated with COVID-19 infection and vaccination.

### Statistical analyses

All statistical analyses were conducted using RStudio (version 1.4.1103; year 2009-2021 edition) on Mac OS Ventura 13.6.1., using the following packages: base (v4.0.0), dplyr (v1.0.8), readr (v2.1.2), tidyr (v1.2.0), lubridate (v1.8.0), broom (v1.5.7), stats (v4.0.0), and boot (v1.3-30). Categorical variables were described using counts and percentages. Continuous variables were described using means and 95% confidence intervals (CIs). Proportions are described using percentages and 95% CIs. Mean menopause symptom scores and 95% CIs are presented for each age group. Prevalence estimates are calculated based on the proportion of patients with the presence of a symptom score ≥1 on the MSQ and for the proportion of patients with COVID-19 infection– and vaccination–associated menstrual disturbance. A bootstrapping technique was used to calculate the 95% CIs for each prevalence estimate, leveraging the boot (v1.3-30) package in R. A total of 1000 bootstrap samples were generated from the original data set, resulting in a distribution of bootstrap prevalence estimates and then used to calculate the 95% CIs of each estimate. A stepwise reverse multivariable linear regression model was used to measure the association of the key predictors with the total MSQ score. The model was initialized using the following predictors: age and age squared (or age group), IMD, clinic location, menstrual status, menstrual regularity, menopause symptom experience, family history of early menopause, hormonal contraception use, presence of a gynecologic diagnosis, COVID-19 infection–associated menstrual disturbance, and COVID-19 vaccination–associated menstrual disturbance. Age was modeled in three ways: age as a continuous numeric variable, age as age squared owing to preexisting parabolic relationships between age and total MSQ score [[17], [18], [19], [20]], and age as a categorical variable of age groups to determine whether there was a statistically significant difference in total MSQ score between age groups. Non-statistically significant predictors were removed (*P* >0.05) individually while assessing for changes in remaining predictor coefficients and corresponding *P*-values with the process repeating until only approximately statistically significant predictors were left. The final models were assessed using clinical judgment, adjusted R^2^, and Akaike information criterion (AIC) to determine model goodness of fit. The initial and final model predictor coefficients, standard error, *P*-value, 95% CIs, adjusted R^2^, and AIC were reported.

## Results

### Summary of cohort

In total, 122 female patients were included in the final cohort after four were excluded for incomplete MSQs (Appendix 3). The age range of recruited patients was 18-72 years and the mean age was 48.1 years (CI 45.9-50.2). The most frequently observed age group was 55-79 years (46 patients; 37.7%), followed by 40-54 years (43 patients; 35.3%), and, finally, 18-39 years (33 patients; 27%). IMD quintile 1 was most frequently observed (37 patients; 31%) and IMD quintile 5 was least frequently observed (9 patients; 7%). Almost 50% of patients lived in the top two most deprived IMD quintiles. Postcode and, therefore, IMD data were missing for nine patients. The majority of patients were seen at the Salford Royal NHS clinic post-COVID syndrome clinic (103 patients; 84%); followed by the Heywood, Middleton, and Rochdale clinic (11 patients; 9%); then the Fairfield clinic (8 patients; 7%).

Of the entire cohort, 55 (45%) patients reported menopause symptom experience (mean age of 55 years; CI 54.0-57.8); 20 (36.4%) patients were in age group 40-54 years and 35 (63.6%) patients in age group 55-79 years. A total of 61 (50%) patients self-reported to menstruate (mean age 40.1 years; CI 38.0-42.3), 56 (46%) patients reported not to menstruate (mean age 57.1 years; CI 54.7-59.5), and 5 (4%) patients were uncertain (mean age 43 years; CI 33.6-52.4). Of patients who reported to experience a menstrual cycle or those who were uncertain, a regular menstrual pattern was most frequently observed (39 patients; 59%) compared with an irregular menstrual pattern (27 patients; 41%).

A menstrual cycle length of 25-30 days was most frequently reported (25 patients; 40%). Furthermore, 15 patients reported a family history of early menopause (12%), 84 patients reported no family history of early menopause (69%), and 23 patients did not know (19%). Only 21 patients (17%) were using contraception, 13 were using hormonal contraception, and 8 were using non-hormonal contraception. A total of 36 patients reported one or more (29.5%) gynecologic diagnoses. The most frequently reported gynecologic diagnosis was endometriosis (11 patients; 9%). The most frequently reported gynecologic surgery was hysterectomy (nine patients; 7.4%) and cesarean section (nine patients; 7.4%).

### MSQ results

#### Summary of individual menopause symptom prevalence for entire cohort

The top five symptoms in order of prevalence were the following (Table 2): 97.5% experienced feeling tired or lacking in energy (CI 94.3-100), 95.9% experienced muscle and joint pains (CI 91.8-99.2), 92.6% experienced memory problems (CI 87.7-96.7), 92.6% experienced difficulty in concentrating (CI 87.7-96.7), and 88.5% experienced feeling tense or nervous (CI 82.0-93.5). The five least prevalent symptoms across the cohort were the following: 34.4% experienced vaginal dryness (CI 27.1-43.4), 45.9% experienced urinary symptoms (CI 36.9-54.9), 58.2% experienced crying spells (CI 50.0-66.4), 62.3% experienced hot flushes (CI 53.3-71.3), and 63.1% experienced loss of interest in sex (CI 54.9-70.5).

**Table 2:** individual menopause symptom questionnaire prevalence (score ≥1) and 95% confidence intervals across entire cohort and age groups.

#### Summary of individual menopause symptom prevalence by age groups

For age group 18-39 years (Table 2), the five most prevalent symptoms were the following: 100% experienced memory problems, 100% experienced feeling tired or lacking in energy, 93.9% experienced feeling tense or nervous (CI 84.9-100), 93.9% experienced difficulty in concentrating (CI 84.9-100), and 93.9% experienced muscle and joint pains (CI 84.9-100). The five least prevalent symptoms were the following: 21.2% experienced vaginal dryness (CI 6.1-36.4), 45.5% experienced urinary symptoms (CI 30.3-60.6), 57.6% experienced loss of interest in sex (CI 42.4-72.7), 60.6% experienced sweating at night (CI 42.5-75.8), and 60.6% experienced hot flushes (CI 45.5-75.8).

For age group 40-54, the five most prevalent symptoms, similarly, were the following: 100% experienced feeling tired or lacking in energy, 97.7% experienced muscle and joint pains (CI 93.0-100), 97.7% experienced difficulty in concentrating (CI 93.0-100), 95.3% experienced difficulty sleeping, and 95.3% experienced headaches (CI 88.4-100). The five least prevalent symptoms were the following: 37.2% experienced vaginal dryness (CI 23.3-51.2), 51.2% experienced urinary symptoms (CI 37.2-65.1), 65.1% experienced crying spells (CI 48.8-79.1), 67.4% experienced sweating at night (CI 51.2-81.4), and 67.4% experienced hot flushes (CI 53.6-81.4).

For age group 55-79 years, the five most prevalent symptoms were the following: 95.7% experienced muscle and joint pains (CI 89.1-100), 93.5% experienced feeling tired or lacking in energy (CI 87.0-100), 87% experienced difficulty in concentrating (CI 76.1-95.7), 84.8% experienced memory problems (CI 73.9-93.5), and 82.6% experienced feeling tense or nervous (CI 69.6-93.5). The five least prevalent symptoms were the following: 41.3% experienced vaginal dryness (CI 26.1-56.5), 41.3% experienced urinary symptoms (CI 28.3-56.5), 47.8% experienced crying spells (CI 32.3-63.0), 52.2% experienced pressure or tightness in the head (CI 37.0-67.4), and 56.5% experienced loss of interest in sex (CI 43.5-71.7).

#### MSQ scores descriptive statistics

Of the 23 questions in the MSQ (Table 3), age group 40-54 years had the highest mean score across 19 questions (heart beating quickly or strongly, feeling tense or nervous, difficulty sleeping, memory problems, attack of anxiety or panic, difficulty concentrating, loss of interest in most things, feeling unhappy or depressed, crying spells, irritability, feeling faint or dizzy, Pressure or tightness in head, headaches, muscle and joint pains, pins and needles in any body part, breathing difficulties, hot flushes, sweating at night, and loss of interest in sex), the highest total MSQ score (65 of a possible 69), highest minimum total MSQ score (9), and the highest mean symptom scores across all domains of the MSQ were the following: psychological (19.5; CI 17.3-21.7), physical (11.3; CI 9.95-12.7), vasomotor (2.60; CI 1.93-3.28), sexual dysfunction (1.49; CI 1.13-1.85), and genitourinary (1.53; CI 1.00-2.07). Furthermore, age group 40-54 years also had the highest mean scores relating to anxiety (11.1; CI 9.89-12.4) and depression (8.37; CI 7.31-9.43). Age group 18-39 years had the highest mean score across 2 questions (feeling tired or lacking in energy, urinary symptoms), whereas age group 55-79 years had the highest mean scores in two questions only (tinnitus, vaginal dryness). After age group 40-54 years, age group 18-39 years had the next highest total mean MSQ score (31.1; CI 26.6-35.5), followed by age group 55-79 years (29.2; CI 25.5-32.9).

**Table 3:** Mean (and 95% CIs) scores for individual menopause symptoms between age groups.

### Linear regression model to predict the total MSQ score

**Figure 1:**
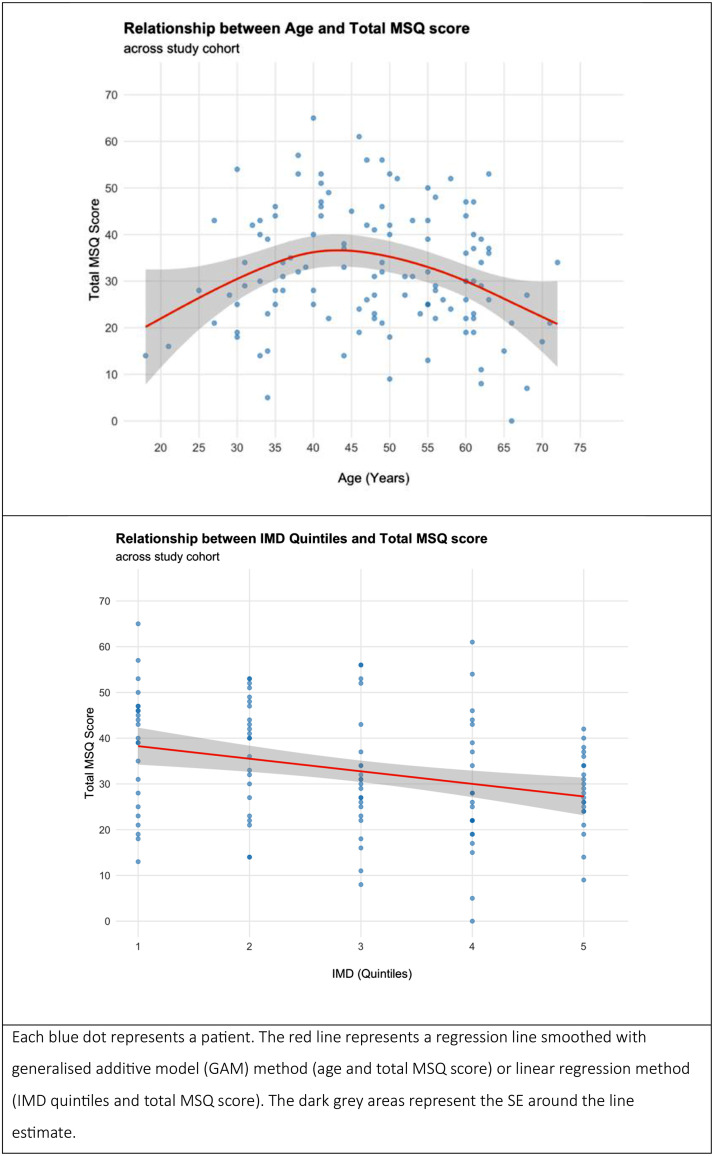
Scatterplots of the (a) total MSQ score across age with regression line and (b) total MSQ score across IMD quintiles with regression line. IMD, index of multiple deprivation; MSQ, menopause symptom questionnaire.

Figure 1a graphically highlights a positive parabolic relationship between age and total MSQ score across the study cohort, increasing from age 18 to approximately age 45 years, where it peaks, with a gradual decline thereafter at a slightly slower rate than the increase. The final model in the multivariable linear regression model (Table 4) quantifies this relationship further. In the final model, age is a significant predictor of total MSQ score; a 1-year increase in age is associated with a 2.13-point increase (CI 0.79 to 3.48) in the total MSQ score (*P* = 0.002), up to a certain point. The negative coefficient for age^2^ (−0.02; CI −0.01 to −0.04) highlights the rate of increase in the total MSQ score slows as age increases (*P* = 0.001). Figure 1b highlights a negative linear relationship between IMD quintile and total MSQ score; an increase in IMD quintile by one level is associated with a 2.67-point lower (CI −1.09 to −4.26) total MSQ score (*P* = 0.001). The presence of any gynecologic diagnosis is associated with a 6.39-point higher (CI 1.49-11.3) total MSQ score (*P* = 0.01). Although not statistically significant, the inclusion of family history of early menopause as a predictor was associated with lower AIC and higher adjusted R^2^ in the final model. After adjusting for predictors, the final model with age modeled as a continuous numeric variable and quadratic (Table 4) explains 20.6% of the variability in the total MSQ score.

**Table 4:** Multivariable linear regression models (initial and final models) – predictors of total menopause symptom questionnaire score (age as a continuous numeric variable and quadratic numeric variable).

	Initial model				Final Model			
	Estimate	SE	*P*-value	95% CIs	Estimate	SE	*P*-value	95% CIs
**Age**								
Age	2.18	0.76	**0.004**	0.67 to 3.69	2.13	0.68	**0.002**	0.79 to 3.48
Age squared	-0.02	0.01	**0.005**	-0.01 to -0.04	-0.02	0.007	**0.001**	-0.01 to -0.04
**Socioeconomic deprivation**								
IMD quintile	-2.50	0.86	**0.004**	-4.20 to 0.81	-2.67	0.80	**0.001**	-1.09 to -4.26
**Clinic location**								
Salford	6.78	5.35	0.21	-3.83 to 17.40	-	-	**-**	-
Fairfield	7.09	7.47	0.34	-7.73 to 21.91	-	-	**-**	-
**Menstruation status**								
Menstruation present	4.18	6.80	0.54	-9.31 to 17.66	-	-	-	-
**Menstrual regularity**								
Regular	-1.68	3.52	0.63	-8.66 to 5.30	-	-	-	-
**Menopause symptom experience**								
Present	0.40	3.01	0.89	-5.57 to 6.37	-	-	-	-
**Family history of early menopause**								
Present	5.86	3.59	0.11	-1.26 to 12.98	5.78	3.37	0.09	-0.91 to 12.5
**Hormonal contraception**								
Present	-1.19	4.54	0.81	-9.90 to 7.64	-	-	-	-
**Gynecological diagnosis**								
Present	6.55	2.61	**0.01**	1.38 to 11.73	6.39	2.47	**0.01**	1.49 to 11.3
**COVID-19 infection–associated menstrual disturbance**								
Present	-3.20	3.16	0.31	-9.46 to 3.07	-	-	-	-
**COVID-19 vaccination–associated menstrual disturbance**								
Present	-0.59	3.72	0.87	-7.96 to 6.78	-	-	-	-

Adjusted R^2^	0.175				0.206			
Akaike information criterion	896.75				884.78			
CI, confidence interval; IMD, index of multiple deprivation; SE, standard error. **Table key**								
*Reference categories*	Age, age squared, and IMD modelled as continuous numerical variables; clinic location – Heywood, Middle, and Rochdale; menstruation status – absence of menstruation; menstrual regularity – irregular menstruation; menopause symptom experience – absent; family history of early menopause – absent; hormonal contraception – absent; gynecological diagnosis – absence of any diagnosis; COVID-19 infection–associated menstrual disturbance – absence; COVID-19 vaccination associated menstrual disturbance – absence.							
*Statistically significant*	values in **bold.**							

When modeling age as age groups (18-39, 40-54, and 55-79 years), the final model (appendix 4) highlights age group 40-54 years as having a significantly higher total MSQ score (6.60 points higher; CI 1.31-11.9) than age group 55-79 years (*P* = 0.01). Furthermore, the final model sees a move toward a borderline significant difference between age group 40-54 and 18-39 years (5.34 points higher for age group 40-54 years; CI 0.38 to −11.1; *P* = 0.06). This model includes similar estimates for IMD and presence of gynecologic diagnosis while observing a move toward significance for the variable of family history of early menopause (presence associated with a 7.04-point higher total MSQ score; CI −0.28 to 13.8; *P* = 0.04). After adjusting for predictors, the final model explains approximately 17.5% of the variability in the total MSQ score.

### Menstrual disturbance with COVID-19 infection and vaccination

In patients who menstruate (n = 61), 50.8% of patients (CI 39.3-62.3) reported COVID-19 infection–related menstrual disturbance (appendix 5), with age group 40-54 years experiencing the highest prevalence (58.8%; CI 41.2-76.4%). Menorrhagia (n = 15) and irregular bleeding (n = 15) were the most frequently reported changes in menstruation, followed by amenorrhea (n = 4), dysmenorrhea (n = 3), and spotting (n = 2). When considering vaccinated patients who menstruate (n = 58), 20.7% (CI 10.3-31.0) reported menstrual disturbance with a COVID-19 vaccine. Menorrhagia (n = 7) and irregular bleeding (n = 3) were most frequently reported menstrual disturbances, followed by dysmenorrhea (n = 1). Of 31 patients who reported menstrual disturbance with COVID-19 infection, 10 (32.3%) patients also reported menstrual disturbance with COVID-19 vaccination.

## Discussion

The primary aim of this study was to measure the menopause symptom prevalence in female patients attending three post-COVID syndrome clinics. Underpinned by a good response rate (72.6%), potential symptoms of perimenopause/menopause as measured using the MSQ were highly prevalent across all three age groups, with patients in age group 40-54 years generally reporting the greatest prevalence of symptoms (Table 2), which correlates clinically with the onset of perimenopause/menopause and is consistent with other research using the Greene Climacteric Scale [17,18,21]. Multivariable regression modeling adds color to this picture, finding age, socioeconomic deprivation as measured using IMD quintiles, and the presence of a gynecologic diagnosis as significant predictors of the total MSQ score.

### Symptom prevalence

Across the cohort, four of the five most prevalent symptoms (Table 2) fell within the psychological domain (feeling tired or lacking in energy) (97.5%; CI 94.3-100), memory problems (92.6%; CI 87.7-96.7), difficulty in concentrating (92.6%; CI 87.7-96.7), and feeling tense or nervous (88.5%; CI 82.0-93.5) of the MSQ. Furthermore, in the physical symptoms domain, the five most prevalent symptoms were muscle and joint pains (95.9%; CI 91.8-99.2), breathing difficulties (84.4%; CI 77.9-91.0), headaches (82.8%; CI 75.4-89.3), feeling dizzy or faint (72.6%; CI 66.6-80.3), and pins and needles in any body part (70.5%; CI 62.3-78.7) (Table 2). Our study generally detects much higher symptom prevalence than three other studies using the Greene Climacteric Scale (appendix 6) [17,18,21]. This may be due to several factors. First, the higher symptom prevalence in our cohort may reflect co-existing and overlapping symptoms of post-COVID syndrome with symptoms of menopause [3]. Second, existing data [17,18,21] do not describe their exact methods for prevalence calculations using MSQs; we calculated the prevalence based on the presence of symptoms based on MSQ score ≥1. Third, cultural factors influence the experience and reporting of menopause symptoms, which may account for variations in the reported prevalence from international studies [17,18,21]. Comparison with observational research from a UK population of 695 women aged 47-54 years shows a peak of severe psychological symptoms within the first years of post-menopause [20], which is partly consistent with higher mean scores in age group 40-54 years in our cohort; however, significant methodologic differences limit further comparisons. Fourth, existing data are from 2004-2019, which covers 2 decades where menopause care and ultimately menopause awareness and acceptance has been suboptimal [10,22].

Classical symptoms of perimenopause and menopause, vasomotor symptoms, were prevalent in approximately two-thirds of patients across the cohort but were in the five least prevalent symptoms overall (Table 2). The overall prevalence of hot flushes (62.3%; CI 53.3-71.3) and sweating at night (63.9%; CI 55.7-73.0) lies within existing ranges in observational research [17,18,21,23]; however, direct comparisons with other cohorts is limited by our analyses being based on age groups (a proxy for reproductive status) as opposed to actual reproductive status. Interestingly, age group 18-39 years also reported a high prevalence of hot flushes (60.6%; CI 45.5-75.8) and sweating at night (60.6%; CI 42.5-75.8), which may seem unusual given that these are often believed to be cardinal symptoms of perimenopause/menopause. However, this finding, albeit to a lesser extent, has been observed before [17,18,21], which may reflect the impact of confounders such as obesity, drugs, and other conditions which cause vasomotor symptoms, including autonomic dysfunction seen in post-COVID syndrome [24,25].

Across the cohort, the prevalence of genitourinary symptoms were the two least prevalent symptoms, with 45.9% of the patients (CI 36.9-54.9) reporting urinary symptoms and 34.4% (CI 27.1-43.4) reporting vaginal dryness. The prevalence estimates observed for urinary symptoms for age groups 40-54 (51.2%; CI 37.2-65.1) and 55-79 years (41.3%; CI 28.3-56.5) and vaginal dryness for age groups 40-54 (37.2%; CI 23.3-51.2) and 55-79 years (41.3%; CI 26.1-56.5) are similar to previous estimates of genitourinary symptoms of menopause from observational research including British (49% prevalence) [26] and other European post-menopausal women (35% prevalence) [27].

In the context of the syndrome of menopause, symptoms were grouped into unifying domains (psychological, physical, vasomotor, sexual, and genitourinary), which supports a clinician's diagnosis and management; in the context of post-COVID syndrome, these symptoms represent vastly different etiologic mechanisms straddling different physiologic domains which must be carefully considered [1]. For example, in perimenopause/menopause, feeling tired or lacking in energy falls within the assessment of symptoms of depression (but may also reflect specific symptoms of sex hormone deficiencies [28]), whereas in post-COVID syndrome, although it could possibly relate to co-existing depression [29], it may be secondary to complex multisystem mechanisms such as but not limited to neuroinflammation, endothelial dysfunction, and microangiopathy [1]. This highlights the need for clinicians to carefully consider all symptoms against preexisting clinical information to inform subsequent diagnoses and management and monitor symptoms in response to treatment. Furthermore, although the review by Atchison et al. [3] is robust, an earlier review [30] identified post-COVID syndrome symptom patterns that also included vasomotor symptoms, indicating a possible underestimation of symptom overlap between the MSQ and those documented by Atchison et al. [3].

### Predictors of total MSQ score

Our study observes a positive parabolic relationship between age and total MSQ score seen at varying degrees in existing research [[17], [18], [19], [20]]. Age group 40-54 years had the highest mean total MSQ score (36.4; CI 32.3-40.6) and the highest mean scores across each domain of the MSQ (Table 3). Furthermore, the statistically significant higher total MSQ score seen in age group 40-54 vs age group 50-79 years and the borderline statistically significant higher total MSQ score seen in age group 40-54 vs age group 18-39 years provide some confidence that the study has detected a true signal of menopause symptom experience in female patients attending post-COVID-19 syndrome clinics.

Where observational research has highlighted varying strengths of association between lifetime economic distress and age of entry into perimenopause [31,32], there is scant research to explore the relationship between socioeconomic deprivation and menopause symptom experience. Our study provides evidence of socioeconomic deprivation (measured using IMD quintiles) as a predictor of menopause symptom experience through its relationship with the total MSQ score; in the final model from Table 4 (highest adjusted R^2^), each increase in IMD quintile was associated with a 2.67-point lower (CI −1.09 to −4.26) total MSQ score. This is partly consistent with other observational research of women in the UK showing that women of a manual social class were more likely to experience severe or very severe symptoms [20]. However, our observed association may be reflecting unmeasured factors known to influence menopause symptom experience, such as but not limited to lower hormone replacement therapy (HRT) prescribing rates in areas of increased deprivation, [33] or may be skewed by the unequal distribution of patients in our study in more deprived IMD quintiles.

In the final model in Table 4, the presence of a gynecologic diagnosis is associated with a 6.39-point significantly higher total MSQ score (CI 1.49-11.3; *P* = 0.01). Although it is unsurprising to find that women with gynecologic diagnoses have higher symptom scores, the inclusion of this variable in the regression model helps to control for its confounding effect in the relationship between age and MSQ score.

Collapsing the presence of all self-reported gynecologic diagnoses into one variable partially obscures its predictive capacity but was necessary to counter the low counts of individual gynecologic diagnoses across the cohort. Nevertheless, its inclusion in the final model is supported by a higher adjusted R^2^ and lower AIC than without it and highlights the need for future modeling to include a larger sample size with a full range of gynecologic diagnoses.

The pathophysiologic mechanisms surrounding early menopause and POI are unclear; however, a positive family history of early menopause or POI carries a six- to eight-fold increased risk of menopause before age 45 years [15]. Interestingly, where age was modeled as age groups (appendix 4), the final model identified a positive family history of early menopause as having borderline significance as a predictor of the total MSQ score (7.04 points higher; CI 0.28-13.8; P = 0.04). We acknowledge that we cannot make any meaningful conclusions from this finding but present it as a point of interest because it has never been shown before [15] and sets the stage for future modeling of family history of early menopause as a predictor of menopause symptom experience and not just as a predictor of early menopause.

It is important to consider that COVID-19 infection–/vaccination–associated menstrual disturbance was not a significant predictor of total MSQ score, which may reflect the limitations of our study design that did not interrogate the temporal relationship between menopause symptom experience and COVID-19 infection/vaccination or possibly reflect short-lived perturbations [4,6] that do not impact the total MSQ score.

### Menstrual disturbance with COVID-19 infection and vaccination

The prevalence of menstrual disturbance with COVID-19 infection in our study is 51% (CI 39.3-62.3), with disturbance of menorrhagia and irregular menstrual bleeding as the most frequently observed. These findings fall within previous estimates of prevalence and type of menstrual disturbance [4,5,[34], [35], [36]] reported after COVID-19 infection. Hypothesized mechanisms for such temporary disturbances include secondary to direct sex organ dysfunction due to increased prevalence of angiotensin-converting enzyme 2 receptors in the ovaries and endometrium and/or systemic immune responses [4,5,9]; however, no study to date has determined the cause [1]. The overall prevalence of menstrual disturbance with COVID-19 vaccination in our study was 21% (CI 10.3-31.0), which is far lower than the estimates from large-scale observational research [37], possibly due to our small sample size and our study relying on self-reported data with fewer exclusions. Of 31 patients who reported menstrual disturbance with acute COVID-19 infection, 32.3% also reported menstrual disturbance with COVID-19 vaccination, a finding which was not previously reported. Ultimately, COVID-19 infection– and vaccination–associated menstrual disturbance create difficulty in designing eligibility criteria that create cohorts based on reproductive status. Future research could exclude patients with COVID-19 infection– and vaccination–associated menstrual disturbance or use sensitivity analyses to address this issue.

### Limitations

Inherent in our study design were the choices to restrict the measurement of menopause symptom experience to those symptoms within the MSQ [7] that overlap with symptoms of post-COVID syndrome, as documented by Atchison et al. [3]. These choices were necessary to provide some constraints around this exploratory research but also mean that we cannot extend our interpretation to the whole spectrum of symptoms widely documented in post-COVID syndrome or additional symptoms in other MSQs. All analyses were based on self-reported data, which are subject to a range of biases (e.g. response bias and availability bias) that can under- and overestimate the prevalence of health status and symptoms. The study sample size was small and not determined by a power calculation but by the availability of patients attending three post-COVID syndrome clinics within a specific timeframe; this resulted in an unequal distribution of patients in each age group and may have reduced our ability to detect statistically significant differences. IMD quintile 1 were overrepresented (31%) in our cohort compared with annual national data for post-COVID syndrome clinics (Appendix 7: 18.2%). Patients were grouped by age groups because COVID-19 infection– and vaccine–associated menstrual disturbance creates challenges in determining which patients are truly perimenopausal; therefore, caution must be exercised in extrapolating findings from age groups to reproductive status. Analyses were not adjusted for current or previous HRT use.

## Conclusion

In conclusion, to the best of our knowledge, this is the first study to measure symptom prevalence, which may be menopausal in origin in female patients attending post-COVID syndrome clinics. Although it is unsurprising to state that some women attending post-COVID syndrome clinics also have symptoms of perimenopause/menopause, it is compelling that our study shows a high prevalence of symptoms in age group 40-54 years consistent with perimenopause/menopause. Moreover, it is also compelling that we find in our cohort the presence of a positive parabolic relationship between menopause symptom experience and age, which is observed to varying degrees in women without post-COVID syndrome [[17], [18], [19], [20]], again, raising the question regarding the origin of these symptoms. Such a finding provides a strong enough signal to stimulate further research into this greatly overlooked area of women's health and post-COVID syndrome research.

Looking ahead, sex disaggregated data are key to accurately comparing patterns of disease prevalence and ultimately interrogating pathophysiologic mechanisms between sexes [9,38]. Greater clarity of prevalence calculation reporting when using MSQs are needed for comparison of menopause symptom experience across populations, systematic reviews, and meta-analyses. In addition, greater baseline measurement of menopause symptom prevalence and pre- and post-intervention with HRT would provide useful comparators for ongoing post-COVID syndrome research. Measurement of clinically verified additional predictors/confounders will further shape our understanding of the temporal relationship between COVID-19 infection/vaccination and post-COVID syndrome and associations with menopause. Study design for future research amidst the evolving landscape of post-COVID syndrome service delivery is important for researchers to consider. Assessment of perimenopause/menopause symptom prevalence in women not attending post-COVID syndrome clinics (i.e., in primary care) would serve as a useful comparator. An interrupted time series could bring interventional level causal effect estimations to cheaper and flexible cohort study designs.

Although our findings are not robust enough to warrant firm recommendations to change clinical practice, the current clinical case definition of post-COVID syndrome supports assessing for other diagnoses before making a diagnosis of post-COVID syndrome [2]; therefore, it is not unreasonable where clinically indicated for clinicians to measure menopause symptom prevalence in women before referral or within post-COVID syndrome clinics and consider a trial of safe and effective HRT.

## Declarations of competing interest

The authors have no competing interests to declare.

## Appendix Supplementary materials

Image, application 1

Image, application 2

Image, application 3

Image, application 4

Image, application 5

Image, application 6

Image, application 7

Image, application 8
