# Supplementary figures and images for: Menopause symptom prevalence in three post–COVID-19 syndrome clinics in England: A cross-sectional analysis
Source: IJID Reg. 2024 Jul 15;12:100405. doi: 10.1016/j.ijregi.2024.100405 (PMC11342884; doi:10.1016/j.ijregi.2024.100405)

## Appendix 1: Women’s health questionnaire


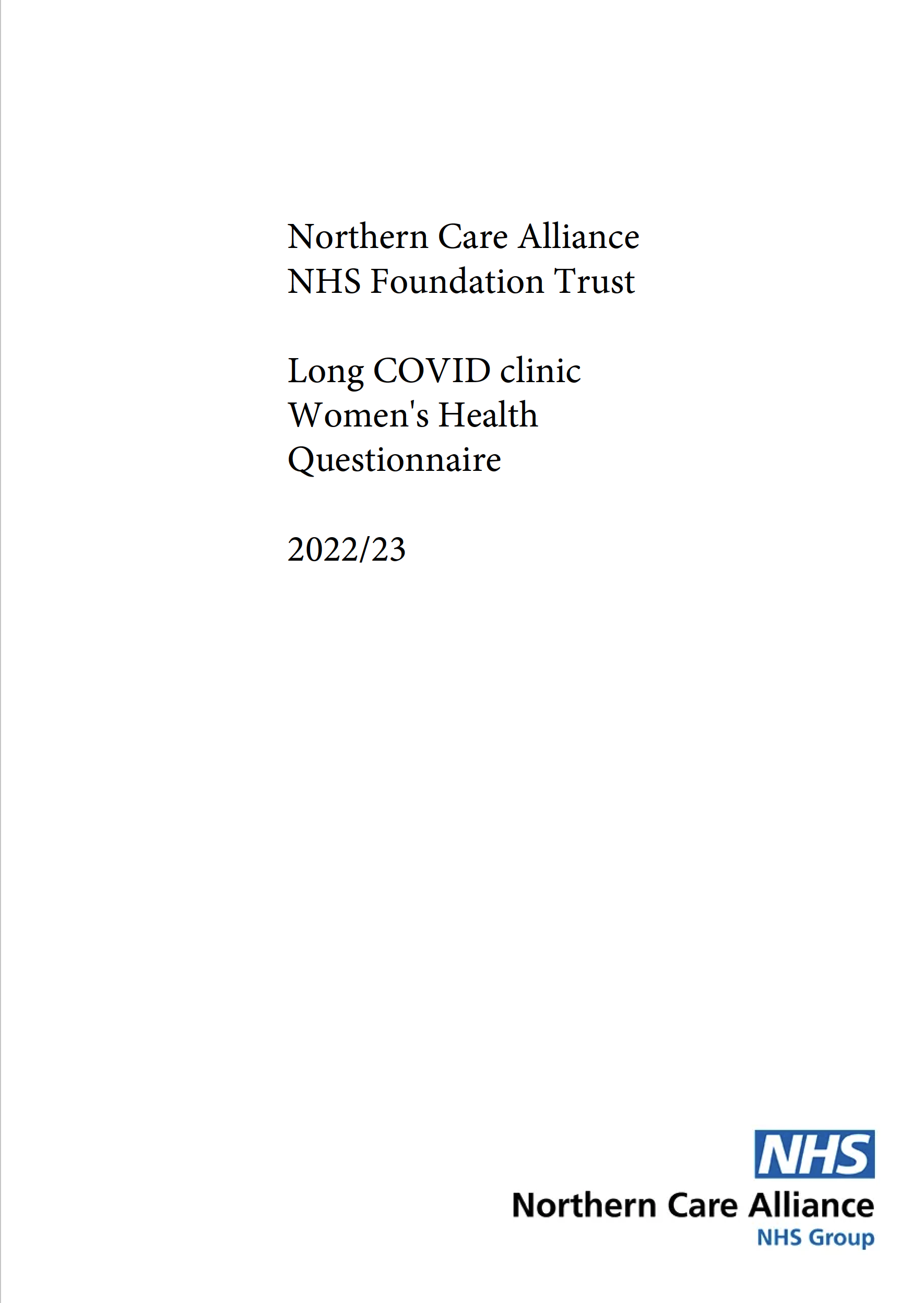


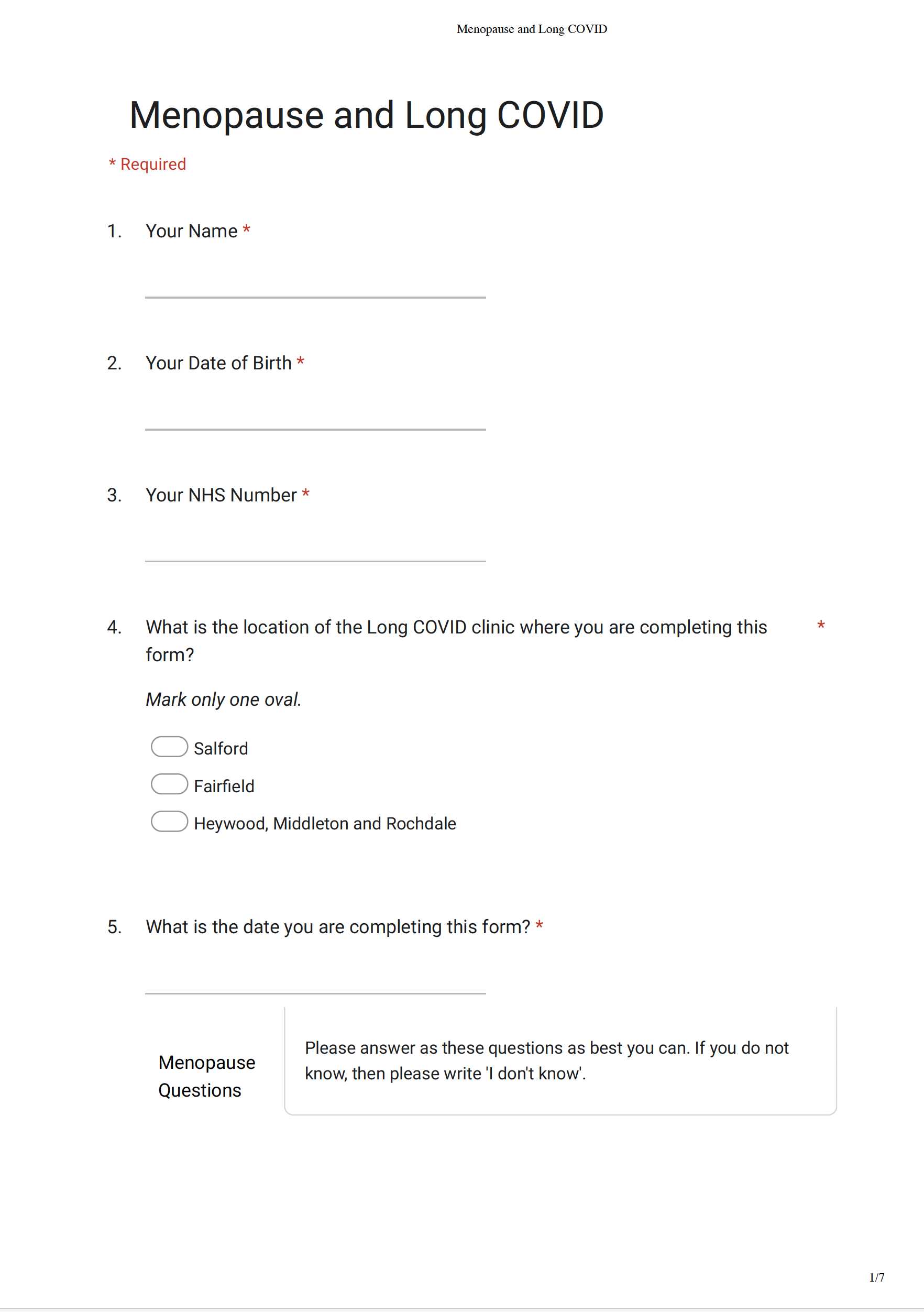


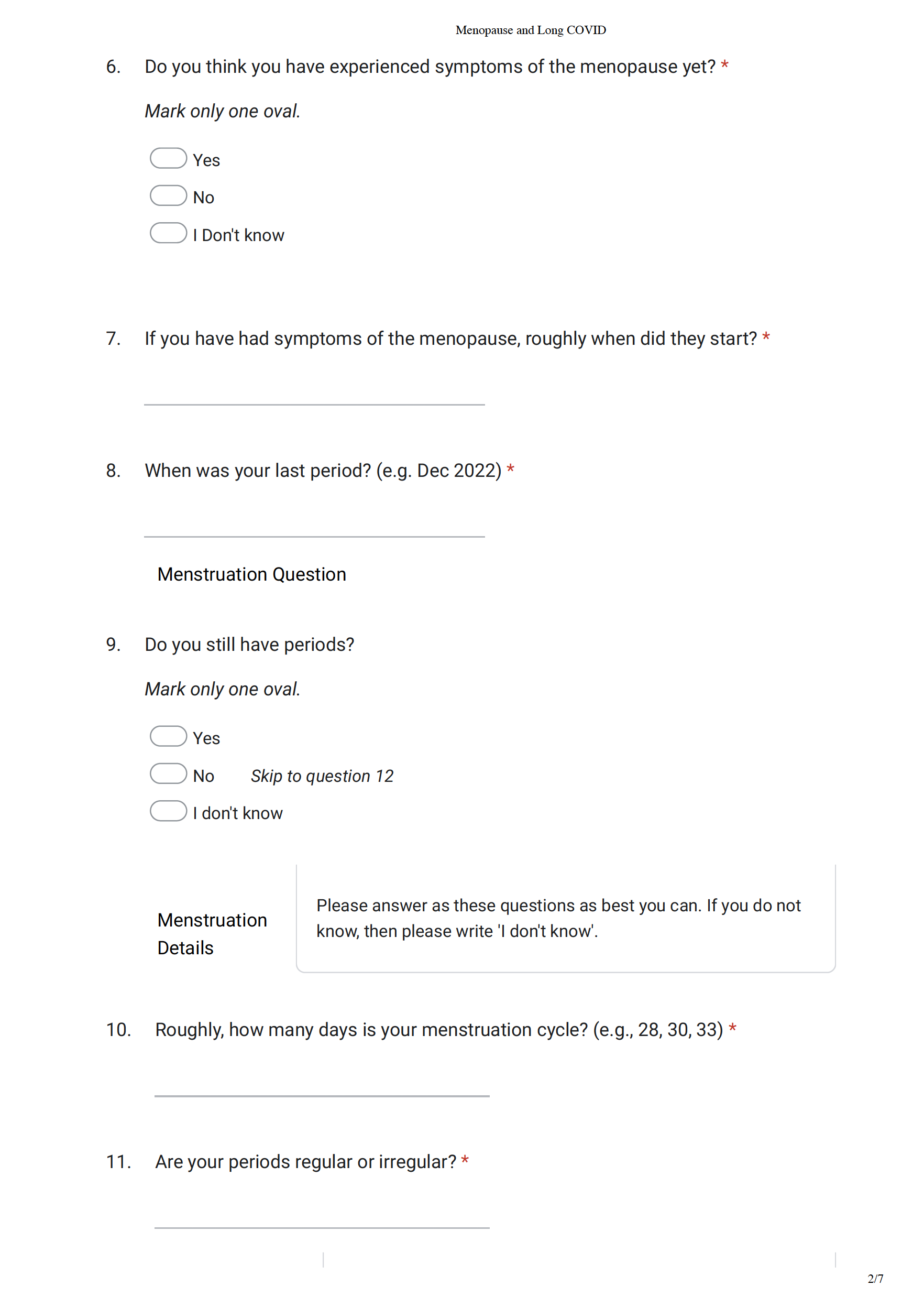


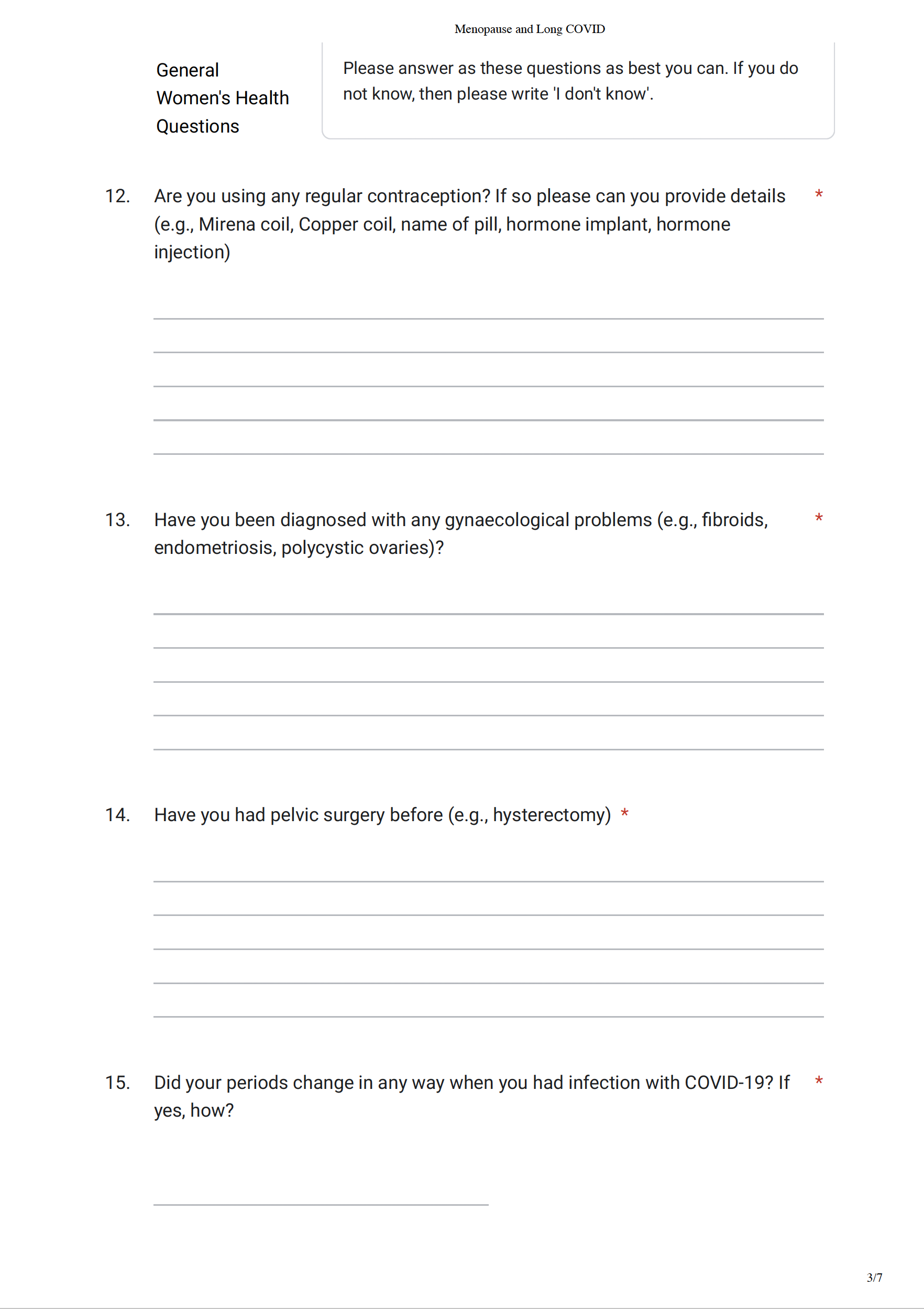


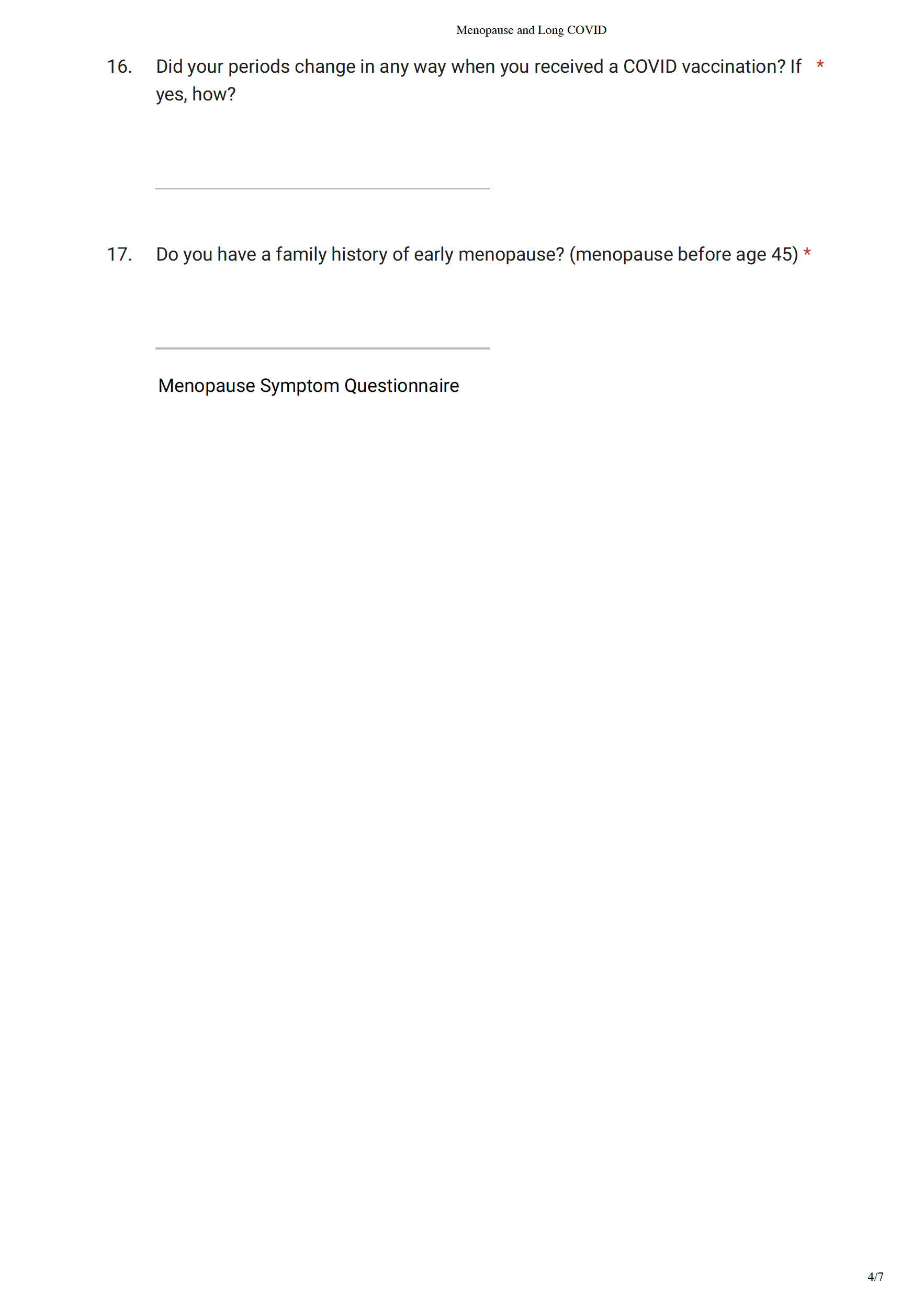


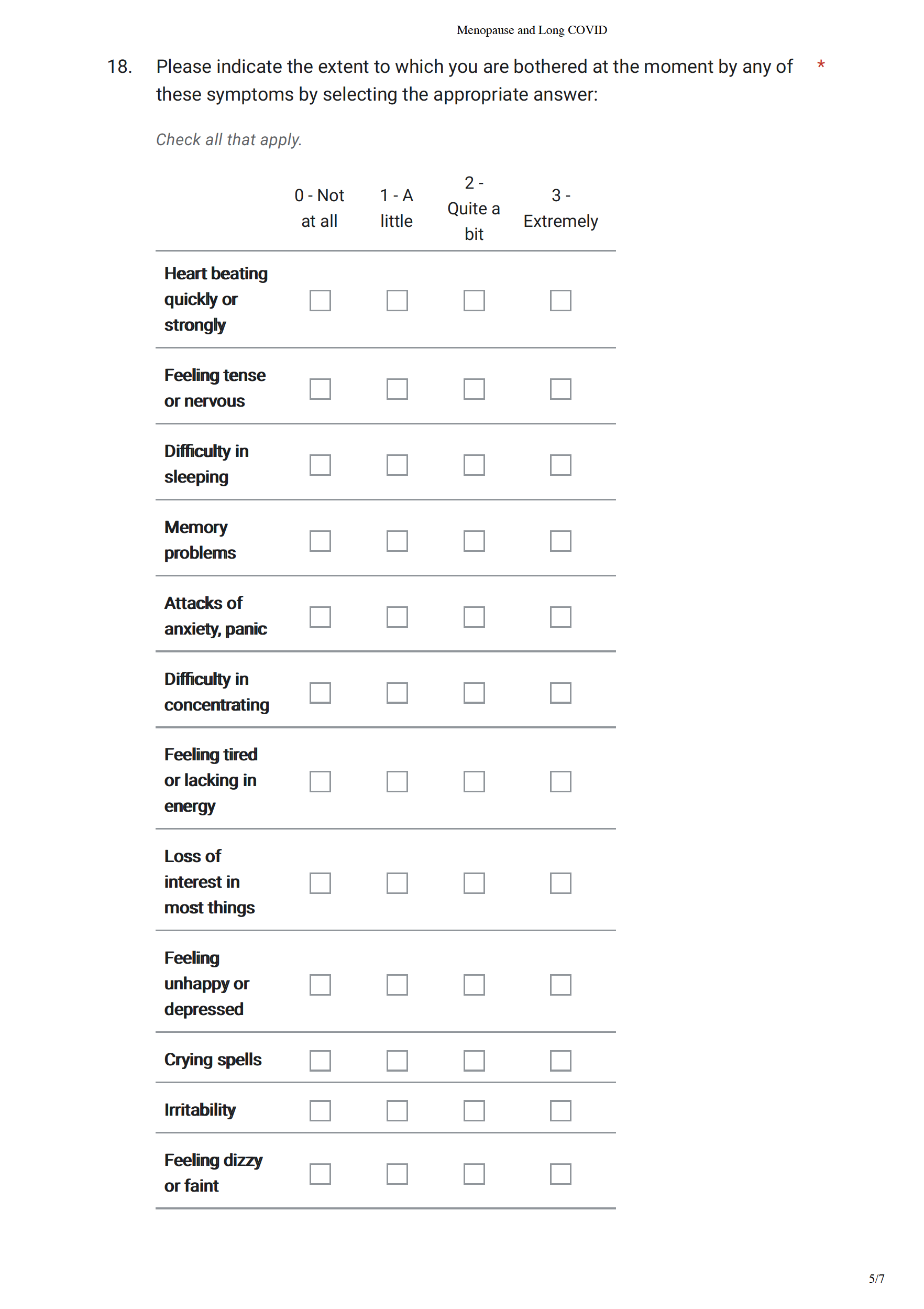


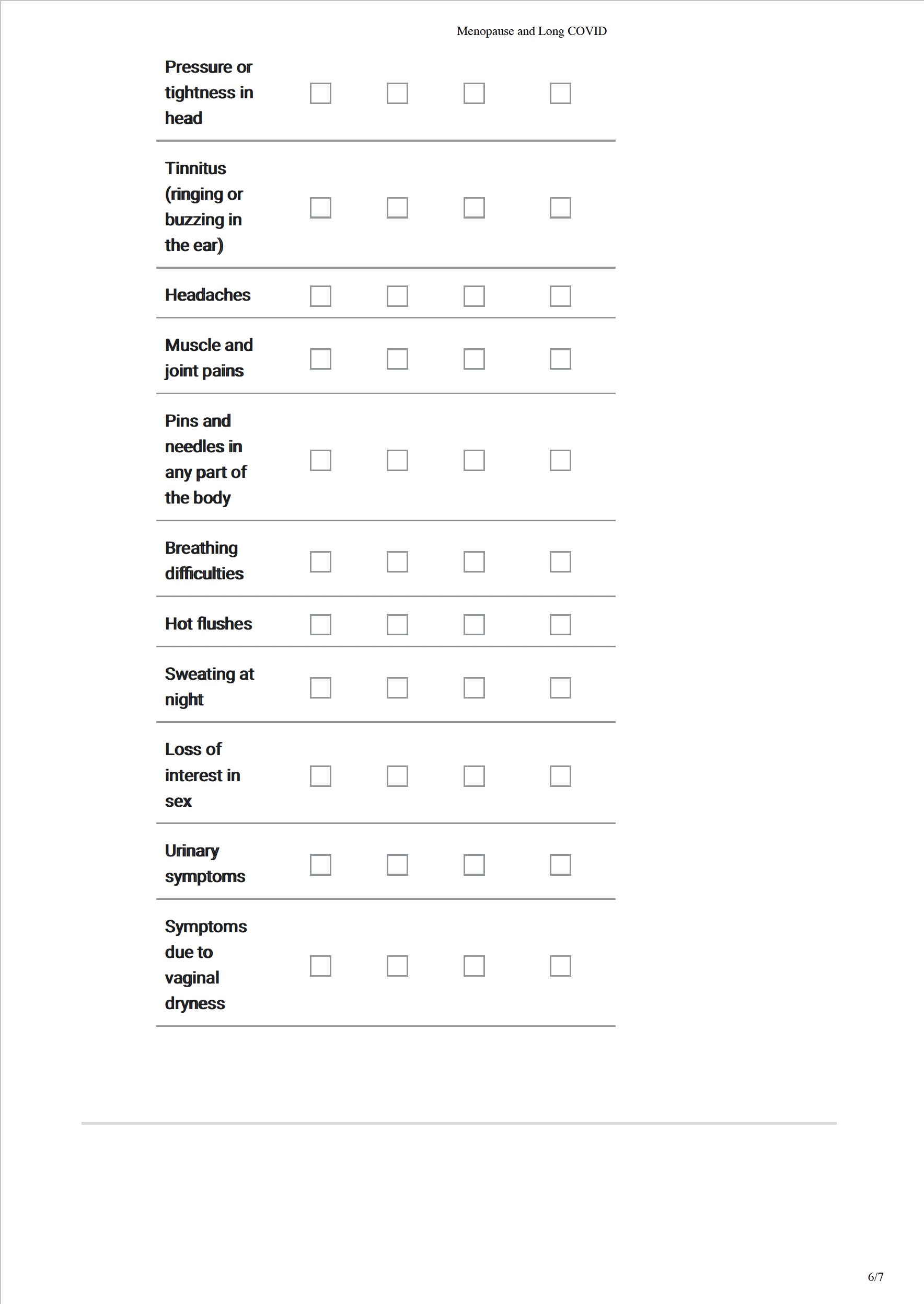

Supplement: Supplementary file 1 [file mmc1.docx]
