# Supplementary material for: Menopause symptom prevalence in three post–COVID-19 syndrome clinics in England: A cross-sectional analysis
Source: IJID Reg. 2024 Jul 15;12:100405. doi: 10.1016/j.ijregi.2024.100405 (PMC11342884; doi:10.1016/j.ijregi.2024.100405)
Supplement: Supplementary file 2 [file mmc2.docx]

## Appendix 2: Data dictionary

**Appendix 2: Data dictionary of all study variables obtained from women’s health questionnaire**

| **Variable name** | **Type** | **Description** | **Units** | **Categories/Values** | **Missing data coding** | **Source** | **Notes** |
| --- | --- | --- | --- | --- | --- | --- | --- |
| Age | Integer | Age of patient | Years | 18-79 | None | Self-reported |  |
| Age squared | Integer | Square root of age | Numeric | 324-5184 | None | Calculated |  |
| Age groups | Categorical | 3 groups | Categories | - 18-39 - 40-54 - 55-79 | None | Calculated |  |
| IMD | Integer | IMD quintile | Numeric | 1-5 | NA | From patient post-code in electronic health record |  |
| Clinic location | Categorical | Clinic name | Categories | - Salford (reference) - Heywood, Middleton and Rochdale - Fairfield | None | Electronic health record |  |
| Menstrual status | Categorical | Self-report of menstrual status | Categories | - Present - Absent - Uncertain | None | Self-reported | ‘Absent’ and ‘Uncertain’ collapsed into one category – ‘Absent’ for modelling |
| Menstrual regularity | Categorical | Self-report of menstrual regularity | Categories | - Regular - Irregular - Not applicable as not menstruating | None | Self-reported |  |
| Menstrual cycle length | Categorical | Self-report of menstrual cycle | Categories | - <10 - 10-14 - 15-20 - 21-24 - 25-30 - 31-35 - 36-50 - >50 | NA | Self-reported |  |
| Menopause symptom experience | Categorical | Self-report of menopause symptom experience | Categories | - Yes - No - I don’t know | None | Self-reported | ‘No’ and ‘I don’t know’ collapsed into one category – ‘No’ for modelling |
| Family history of early menopause | Categorical | Self-report of family history of early menopause | Categories | - Yes - No - I don’t know | None | Self-reported | ‘No’ and ‘I don’t know’ collapsed into one category – ‘No’ for modelling |
| Contraception use | Categorical | Current contraception use | Categories | - Yes - No | No | Self-reported |  |
| Type of Contraception | Categorical | Current type of contraception use | Categories | - Progesterone only pill - Combined oral contraceptive pill - Intrauterine device (non-hormonal) - Intrauterine system (hormonal) - Sterilisation | None | Self-reported |  |
| Hormonal contraception | Categorical | Current hormonal contraception use | Categories | - Absent - Present | None | Self-reported | Present if POP, COCP, IUS present |
| Gynaecological diagnosis | Categorical | Presence of any gynaecological diagnosis | Categories | - Absent - Present | None | Self-reported | Present if any recorded gynaecological diagnosis self-reported. |
| Endometriosis | Categorical | Presence of condition | Categories | - Absent - Present | None | Self-reported |  |
| Hysterectomy | Categorical | Presence of condition | Categories | - Absent - Present | None | Self-reported |  |
| Caesarean section | Categorical | Presence of condition | Categories | - Absent - Present | None | Self-reported |  |
| Fibroids | Categorical | Presence of condition | Categories | - Absent - Present | None | Self-reported |  |
| Polycystic ovarian syndrome | Categorical | Presence of condition | Categories | - Absent - Present | None | Self-reported |  |
| Pelvic organ prolapse | Categorical | Presence of condition | Categories | - Absent - Present | None | Self-reported |  |
| Ovarian cysts | Categorical | Presence of condition | Categories | - Absent - Present | None | Self-reported |  |
| Adenomyosis | Categorical | Presence of condition | Categories | - Absent - Present | None | Self-reported |  |
| Gynaecological cancer | Categorical | Presence of condition | Categories | - Absent - Present | None | Self-reported |  |
| Sterilisation | Categorical | Presence of condition | Categories | - Absent - Present | None | Self-reported |  |
| Menorrhagia | Categorical | Presence of condition | Categories | - Absent - Present | None | Self-reported |  |
| COVID-19 infection menstrual disturbance | Categorical | Self-report of any COVID-19 infection associated menstrual disturbance | Categories | - Present - Absent | None | Self-reported |  |
| COVID-19 infection menorrhagia | Categorical | Self-report of any COVID-19 infection associated menstrual disturbance | Categories | - Present - Absent | None | Self-reported |  |
| COVID-19 infection amenorrhoea | Categorical | Self-report of any COVID-19 infection associated menstrual disturbance | Categories | - Present - Absent | None | Self-reported |  |
| COVID-19 infection dysmenorrhoea | Categorical | Self-report of any COVID-19 infection associated menstrual disturbance | Categories | - Present - Absent | None | Self-reported |  |
| COVID-19 infection spotting | Categorical | Self-report of any COVID-19 infection associated menstrual disturbance | Categories | - Present - Absent | None | Self-reported |  |
| COVID-19 infection irregular bleeding | Categorical | Self-report of any COVID-19 infection associated menstrual disturbance | Categories | - Present - Absent | None | Self-reported |  |
| COVID-19 vaccination menstrual disturbance | Categorical | Self-report of any COVID-19 vaccination associated menstrual disturbance | Categories | - Present - Absent | None | Self-reported |  |
| COVID-19 vaccination menorrhagia | Categorical | Self-report of any COVID-19 vaccination associated menstrual disturbance | Categories | - Present - Absent | None | Self-reported |  |
| COVID-19 vaccination amenorrhoea | Categorical | Self-report of any COVID-19 vaccination associated menstrual disturbance | Categories | - Present - Absent | None | Self-reported |  |
| COVID-19 vaccination dysmenorrhoea | Categorical | Self-report of any COVID-19 vaccination associated menstrual disturbance | Categories | - Present - Absent | None | Self-reported |  |
| COVID-19 vaccination spotting | Categorical | Self-report of any COVID-19 vaccination associated menstrual disturbance | Categories | - Present - Absent | None | Self-reported |  |
| COVID-19 vaccination irregular bleeding | Categorical | Self-report of any COVID-19 vaccination associated menstrual disturbance | Categories | - Present - Absent | None | Self-reported |  |
