# Supplementary material for: Menopause symptom prevalence in three post–COVID-19 syndrome clinics in England: A cross-sectional analysis
Source: IJID Reg. 2024 Jul 15;12:100405. doi: 10.1016/j.ijregi.2024.100405 (PMC11342884; doi:10.1016/j.ijregi.2024.100405)
Supplement: Supplementary file 3 [file mmc3.docx]

## Appendix 3: Cohort clinical and demographic summary

**Appendix 3: Cohort clinical and demographic summary**

|  | n (%) |  | n (%) |
| --- | --- | --- | --- |
| Age groups |  | **Menstruation cycle length (days)** |  |
| 18-39 | 33 (27) | <10 | 9 (14) |
| 40-54 | 43 (35.3) | 10-14 | 0 (0) |
| 55-79 | 46 (37.7) | 15-20 | 0 (0) |
|  |  | 21-24 | 2 (3) |
| IMD quintiles |  | 25-30 | 25 (40) |
| 1 (most deprived) | 37 (31) | 31-35 | 3 (5) |
| 2 | 35 (28) | 36-50 | 0 (0) |
| 3 | 19 (16) | >50 | 1 (2) |
| 4 | 13 (11) | Did not state cycle length | 21 (33) |
| 5 | 9 (7) |  |  |
| Missing | 9 (7) | **Menstruation regularity** |  |
|  |  | Regular | 39 (59) |
| Clinic Location |  | Irregular | 27 (41) |
| Fairfield | 8 (7) | Not applicable as menstruation absent | 56 |
| Heywood, Middleton, and Rochdale | 11 (9) |  |  |
| Salford | 103 (84) | **Contraception use** |  |
|  |  | Yes | 21 (17) |
| Experience of menopause symptoms |  | No | 101 (83) |
| Yes | 55 (45) |  |  |
| No | 41 (34) | **Contraception type** |  |
| I don’t know | 26 (21) | Progesterone only pill | 7 (33) |
|  |  | Intrauterine device | 6 (29) |
| Family history of early menopause |  | Intrauterine system | 5 (24) |
| Yes | 15 (12) | Sterilisation | 2 (10) |
| No | 84 (69) | Combined (oestrogen and progesterone) )oral contraceptive pill | 1 (5) |
| I don’t know | 23 (19) |  |  |
|  |  | **Gynaecological diagnosis** |  |
| Menstruation status |  | Present | 36 (29.5) |
| Menstruation present | 61 (50) | Absent | 86 (70.5) |
| Menstruation absent | 56 (46) |  |  |
| Menstruation uncertain | 5 (4) | **Gynaecological conditions or surgery (past history) *** |  |
|  |  | Endometriosis | 11 (9.0) |
|  |  | Hysterectomy | 9 (7.4) |
|  |  | Caesarean section | 9 (7.4) |
|  |  | Fibroids | 9 (7.4) |
|  |  | Polycystic ovarian syndrome | 6 (4.9) |
|  |  | Pelvic organ prolapse | 5 (4.1) |
|  |  | Ovarian cysts | 4 (3.3) |
|  |  | Adenomyosis | 3 (2.5) |
|  |  | Gynaecological cancer | 2 (1.6) |
|  |  | Sterilisation | 2 (1.6) |
|  |  | Menorrhagia | 2 (1.6) |
| Key | | | |
| * Patients may report more than 1 option | | | |
