# Supplementary material for: Menopause symptom prevalence in three post–COVID-19 syndrome clinics in England: A cross-sectional analysis
Source: IJID Reg. 2024 Jul 15;12:100405. doi: 10.1016/j.ijregi.2024.100405 (PMC11342884; doi:10.1016/j.ijregi.2024.100405)
Supplement: Supplementary file 4 [file mmc4.docx]

## Appendix 4: Linear regression model with age as a categorical variable

**Appendix 4: Multivariable linear regression models (initial and final models) – predictors of total MSQ score (age by age groups)**

|  | **Initial model** | | | | **Final Model** | | | |
| --- | --- | --- | --- | --- | --- | --- | --- | --- |
|  | **Estimate** | **SE** | **p-value** | **95% CIs** | **Estimate** | **SE** | **p-value** | **95% CIs** |
| **Age Groups** | | | | | | | | |
| 18-39 | -5.84 | 3.34 | 0.08 | -12.45 to 0.78 | -5.34 | 2.89 | 0.06 | -11.1 to 0.38 |
| 55-79 | -5.42 | 4.15 | 0.19 | -13.64 to 2.81 | -6.60 | 2.68 | **0.01** | -1.31 to-11.9 |
| **Socioeconomic deprivation** | | | | | | | | |
| IMD quintile | -2.73 | 0.87 | **0.002** | -1.01 to -4.46 | -2.85 | 0.81 | **0.0006** | -1.24 to-4.45 |
| **Clinic location** | | | | | | | | |
| Salford | 5.79 | 5.41 | 0.29 | -4.96 to 16.53 | - | - | **-** | - |
| Fairfield | 7.27 | 7.65 | 0.34 | -7.91 to 22.45 | - | - | **-** | - |
| **Menstruation status** | | | | | | | | |
| Menstruation present | 2.59 | 7.00 | 0.71 | -11.30 to 16.47 | - | - | - | - |
| **Menstrual regularity** | | | | | | | | |
| Regular | -0.49 | 3.57 | 0.89 | -7.56 to 6.59 | - | - | - | - |
| **Menopause symptom experience** | | | | | | | | |
| Present | 1.01 | 3.10 | 0.75 | -5.14 to 7.15 | - | - | - | - |
| **Family history of early menopause** | | | | | | | | |
| Present | 7.01 | 3.61 | 0.06 | -0.16 to 14.18 | 7.04 | 3.41 | **0.04** | 0.28 to 13.8 |
| **Hormonal contraception** | | | | | | | | |
| Present | -1.84 | 4.86 | 0.78 | -11.0 to 7.1 | - | - | - | - |
| **Gynaecological diagnosis** | | | | | | | | |
| Present | 6.56 | 2.66 | **0.02** | 1.28 to 11.85 | 6.31 | 2.51 | **0.01** | 1.32 to 11.3 |
| **COVID-19 infection associated menstrual disturbance** | | | | | | | | |
| Present | -3.28 | 3.23 | 0.31 | -9.69 to 3.14 | - | - | - | - |
| **COVID-19 vaccination associated menstrual disturbance** | | | | | | | | |
| Present | -2.35 | 3.72 | 0.53 | -9.72 to 5.02 | - | - | - | - |
|  | | | | | | | | |
| Adjusted R^2^ | 0.138 | | | | 0.175 | | | |
| AIC | 901.16 | | | | 889.01 | | | |
| **Table Key** | | | | | | | | |
| *Reference categories* | Age modelled as categorical variable – reference group is 40-54; IMD modelled as continuous numerical variables; Clinic location – Heywood, Middle and Rochdale; Menstruation status – absence of menstruation; Menstrual regularity – irregular menstruation; Menopause symptom experience – absent; family history of early menopause – absent; hormonal contraception – absent; Gynaecological diagnosis – absence of any diagnosis; COVID-19 infection associated menstrual disturbance – absence; COVID-19 vaccination associated menstrual disturbance – absence. | | | | | | | |
| *Statistically significant* | values in **bold.** | | | | | | | |
