# Supplementary material for: Menopause symptom prevalence in three post–COVID-19 syndrome clinics in England: A cross-sectional analysis
Source: IJID Reg. 2024 Jul 15;12:100405. doi: 10.1016/j.ijregi.2024.100405 (PMC11342884; doi:10.1016/j.ijregi.2024.100405)
Supplement: Supplementary file 5 [file mmc5.docx]

## Appendix 5: Menstrual disturbance with COVID-19 infection and vaccination

**Appendix 5: Prevalence of menstrual disturbance with COVID-19 infection and COVID-19 vaccination**

|  | |  | Age groups | | |
| --- | --- | --- | --- | --- | --- |
|  | | Entire cohort: menstruation present  n=61 | 18-39  n=26 | 40-54  n=34 | 55-79  n=1 |
|  |  | % (95% CIs) | | | |
| Menstrual change with COVID-19 infection | Present | n=31  50.8 (39.3-62.3) | n=11  42.3 (23.1-61.5) | n=20  58.8 (41.2-76.4) | n=0  0 |
| Types of  menstrual disturbance | Menorrhagia | 15 | 2 | 12 | 1 |
|  | Amenorrhoea | 4 | 2 | 2 | 0 |
|  | Dysmenorrhoea | 3 | 1 | 2 | 0 |
|  | Spotting | 2 | 1 | 1 | 0 |
|  | Irregular bleeding | 15 | 7 | 7 | 1 |
|  | | | | | |
|  | | Entire cohort: menstruation present and vaccinated  n=58 | 18-39  n=24 | 40-54  n=33 | 55-79  n=1 |
|  |  | % (95% CIs) | | | |
| Menstrual change with COVID-19 vaccination | Present | n=12  20.7 (10.3-31.0) | n=3  12.5 (0.0-25.0) | n=8  24.2 (12.1-39.4) | n=1*  100 (100) |
| Types of  menstrual disturbance | Menorrhagia | 7 | 2 | 5 | 0 |
|  | Amenorrhoea | 0 | 0 | 0 | 0 |
|  | Dysmenorrhoea | 1 | 1 | 0 | 0 |
|  | Spotting | 0 | 0 | 0 | 0 |
|  | Irregular bleeding | 3 | 0 | 3 | 0 |
| *1 patient did not describe their menstrual disturbance with COVID-19 vaccination | | | | | |
