# Supplementary material for: Menopause symptom prevalence in three post–COVID-19 syndrome clinics in England: A cross-sectional analysis
Source: IJID Reg. 2024 Jul 15;12:100405. doi: 10.1016/j.ijregi.2024.100405 (PMC11342884; doi:10.1016/j.ijregi.2024.100405)
Supplement: Supplementary file 6 [file mmc6.docx]

## Appendix 6: Menopause symptom prevalence compared with existing evidence

**Appendix 6: Comparison of cohort MSQ scores and 3 Women’s health studies**[16], [17], [20]**.**

|  | Our  Study  n=122 | Anderson  et al[17] Australian  women  n=712 | Anderson  et al[17] Japanese women  n=1430 | Thakur  et al[16] Indian women  n=351 | Sierra et al[20]  Ecuadorian  women  n=385 |
| --- | --- | --- | --- | --- | --- |
| Symptoms | % (95% CIs) | | | | |
| 1. Heart beating quickly or strongly | 71.3 (63.1-79.5) | 35.2 | 51.7 | 47 | 57.1 |
| 2. Feeling tense or nervous | 88.5 (82.0-93.5) | 56.6 | 65.5 | 51 | 74.2 |
| 3. Difficulty sleeping | 86.9 (80.3-92.6) | 65.1 | 46.9 | 56 | 47.8 |
| 4. Memory problems | 92.6 (87.7-96.7) | - | - | - | - |
| 5. Attack of anxiety or panic | 79.5 (72.1-86.1) | 26.7 | 30.7 | 34 | 16.1 |
| 6. Difficulty in concentrating | 92.6 (87.7-96.7) | 59.1 | 76.2 | 49 | 87 |
| 7. Feeling tired or lacking in energy | 97.5 (94.3-100) | - | - | 69 | 77.1 |
| 8. Loss of interest in most things | 77.0 (68.9-83.6) | 33.9 | 55.1 | 54 | 66.9 |
| 9. Feeling unhappy or depressed | 74.6 (66.4-82.8) | 46.4 | 58.8 | 59 | 82 |
| 10. Crying spells | 58.2 (50.0-66.4) | 27.7 | 26.0 | 53 | 81.5 |
| 11. Irritability | 83.6 (76.2-90.2) | 55.6 | 52.2 | 59 | 28.5 |
| 12. Feeling dizzy or faint | 72.6 (66.6-80.3) | 23.9 | 57.1 | 30 | 56.1 |
| 13. Pressure or tightness in head | 63.9 (54.9-72.3) | - | - | 38 | 29.6 |
| 14. Tinnitus | 64.8 (56.6-73.0) | - | - | - | - |
| 15. Headaches | 82.8 (75.4-89.3) | 53.6 | 60.6 | 44 | 83.9 |
| 16. Muscle and joint pains | 95.9 (91.8-99.2) | 77.9 | 70.4 | 50 | 79.5 |
| 17. Pins and needles in hands and feet | 70.5 (62.3-78.7) | - | - | 29 | 37.9 |
| 18. Breathing difficulties | 84.4 (77.9-91.0) | - | - | 28 | 19.5 |
| 19. Hot flushes | 62.3 (53.3-71.3) | 45.3 | 46.0 | 28 | 82 |
| 20. Sweating at night | 63.9 (55.7-73.0) | 38.8 | 29.2 | 37 | 65.7 |
| 21. Loss of interest in sex | 63.1 (54.9-70.5) | 70.4 | 71.5 | 83 | 75.8 |
| 22. Urinary symptoms | 45.9 (36.9-54.9) | - | - | - | - |
| 23. Vaginal dryness | 34.4 (27.1-43.4) | - | _-_ | _-_ | _-_ |
