# Supplementary material for: Menopause symptom prevalence in three post–COVID-19 syndrome clinics in England: A cross-sectional analysis
Source: IJID Reg. 2024 Jul 15;12:100405. doi: 10.1016/j.ijregi.2024.100405 (PMC11342884; doi:10.1016/j.ijregi.2024.100405)
Supplement: Supplementary file 7 [file mmc7.docx]

## Appendix 7: Post-COVID syndrome clinic activity and demographic summary in North West England

**Appendix 7: Post-COVID syndrome clinic data in North West England From October 2022 to September 2023 (redrawn from NHS England data)**[38]

|  | Initial specialist assessments  n | Male sex  n | Female sex  n | Age >= 45  n | IMD 1-2  n | IMD 3-10  n | England IMD 1-2  n |
| --- | --- | --- | --- | --- | --- | --- | --- |
| September 2023 | 257 | 85 | 172 | 184 | 77 | 179 | 17.5% |
| August 2023 | 242 | 84 | 157 | 167 | 75 | 166 | 16.4% |
| July 2023 | 290 | 105 | 185 | 216 | 94 | 195 | 17.4% |
| June 2023 | 298 | 95 | 203 | 204 | 75 | 223 | 16.8% |
| May 2023 | 266 | 77 | 189 | 188 | 89 | 177 | 18.3% |
| April 2023 | 240 | 78 | 162 | 157 | 79 | 161 | 19.7% |
| March 2023 | 289 | 87 | 196 | 207 | 113 | 170 | 18.8% |
| February 2023 | 331 | 92 | 239 | 222 | 133 | 200 | 18.5% |
| January 2023 | 381 | 131 | 243 | 277 | 123 | 258 | 17.8% |
| December 2022 | 262 | 103 | 159 | 185 | 67 | 195 | 16.6% |
| November 2022 | 330 | 109 | 221 | 215 | 118 | 212 | 19.4% |
| October 2022 | 380 | 132 | 247 | 268 | 129 | 251 | 21.7% |
| Annual total | 3566 | 1178 (33%) | 2373  (67%) | 2390  (67%) | 1172 (33%) | 2387 (67%) | 18.24% |
| Absolute counts are presented with percentages in brackets (except for final column) | | | | | | | |
