# Supplementary material for: Menopause symptom prevalence in three post–COVID-19 syndrome clinics in England: A cross-sectional analysis
Source: IJID Reg. 2024 Jul 15;12:100405. doi: 10.1016/j.ijregi.2024.100405 (PMC11342884; doi:10.1016/j.ijregi.2024.100405)
Supplement: Supplementary file 8 [file mmc8.docx]

**Caption for supplementary material**

1. Appendix 1: Northern Care Alliance NHS foundation trust women’s health questionnaire including the Balance MSQ

2. Appendix 2: Data dictionary

3. Appendix 3: Cohort clinical and demographic summary

4. Appendix 4: Linear regression model with age as a categorical variable

5. Appendix 5: Menstrual disturbance with COVID-19 infection and vaccination

6. Appendix 6: Menopause symptom prevalence compared with existing evidence

7. Appendix 7: Post-COVID syndrome clinic activity and demographic summary in North West England
